# On the Zero-Outage Secrecy-Capacity of Dependent Fading Wiretap Channels

**DOI:** 10.3390/e24010099

**Published:** 2022-01-08

**Authors:** Eduard Jorswieck, Pin-Hsun Lin, Karl-Ludwig Besser

**Affiliations:** Institute for Communications Technology, TU Braunschweig, 38106 Braunschweig, Germany; Lin@ifn.ing.tu-bs.de (P.-H.L.); besser@ifn.ing.tu-bs.de (K.-L.B.)

**Keywords:** wiretap channel, secrecy capacity, dependent fading, copulas

## Abstract

It is known that for a slow fading Gaussian wiretap channel without channel state information at the transmitter and with statistically independent fading channels, the outage probability of any given target secrecy rate is non-zero, in general. This implies that the so-called zero-outage secrecy capacity (ZOSC) is zero and we cannot transmit at any positive data rate reliably and confidentially. When the fading legitimate and eavesdropper channels are statistically dependent, this conclusion changes significantly. Our work shows that there exist dependency structures for which positive zero-outage secrecy rates (ZOSR) are achievable. In this paper, we are interested in the characterization of these dependency structures and we study the system parameters in terms of the number of observations at legitimate receiver and eavesdropper as well as average channel gains for which positive ZOSR are achieved. First, we consider the setting that there are two paths from the transmitter to the legitimate receiver and one path to the eavesdropper. We show that by introducing a proper dependence structure among the fading gains of the three paths, we can achieve a zero secrecy outage probability (SOP) for some positive secrecy rate. In this way, we can achieve a non-zero ZOSR. We conjecture that the proposed dependency structure achieves maximum ZOSR. To better understand the underlying dependence structure, we further consider the case where the channel gains are from finite alphabets and systematically and globally solve the ZOSC. In addition, we apply the rearrangement algorithm to solve the ZOSR for continuous channel gains. The results indicate that the legitimate link must have an advantage in terms of the number of antennas and average channel gains to obtain positive ZOSR. The results motivate further studies into the optimal dependency structures.

## 1. Introduction

With the rise of new technologies and applications, e.g., in the context of 6G [1], more sensitive data are transmitted wirelessly. Thus, it is crucial to ensure a confidential transmission and protect the information against possible eavesdroppers. Besides cryptography, physical layer security [2] is a promising approach to enable secure data transmission. In contrast to cryptography, no shared key is required, but the physical properties of the wireless channel are exploited. This establishes post-quantum security by information-theoretic methods and offers better protection than many common cryptography schemes, which rely on the complexity of certain computations, that can be solved fast on quantum computers [3].

It is well-known that there exists a certain class of channel codes, which allow transmitting with zero information leakage over the standard additive white Gaussian noise (AWGN) channel. In the case that the channels to the legitimate receiver and the eavesdropper experience fading, it is possible that secrecy outages occur due to the random nature of the channel fading [4]. For slow-fading channels, the appropriate performance metrics are the (secrecy) outage probability and the ε-(secrecy)-outage capacity, i.e., the maximum rate at which one can communicate with at most ε (secrecy) outages.

In order to improve the reliability, multiple antennas are often employed at the receiver or transmitter. Spatial diversity not only improves reliability, but can also be applied at the eavesdropper to increase the information leakage. It heavily depends on the joint distributions of the fading channels, whether benefits in terms of reliability or in terms of information leakage can be achieved. While the marginal fading distributions of the individual fading links can be measured easily, the joint distribution is typically unknown. A common assumption in the literature is, therefore independence [5,6,7], which can have a major impact on the estimated performance [8]. If we consider the standard wiretap channel and consider the secrecy capacity, it is shown in [9] that the ergodic secrecy capacity only depends on the marginal distributions of the observations at Bob and Eve. In contrast, the outage secrecy capacity depends on the joint distribution and the same marginal property does not hold. Additionally, real measurements demonstrate that this assumption does not always hold in practice [10,11]. Especially for physical layer security systems, this can have significant consequences.

It is therefore of great interest to analyze the performance of such communication systems where independence between the marginal fading links does not hold. In [12,13], bounds on the secrecy capacity for correlated Rayleigh fading channels are derived. Similarly, the secrecy performance for correlated log-normal and Gamma fading channels is considered in [14,15], respectively. In all of this previous work, only (positive) linear correlation is considered. However, the joint fading distribution might have nonlinear dependency features [10]. A more general approach is therefore taken in [16], where the secrecy outage probability (SOP) is derived for Rayleigh fading links that follow a certain dependency structure. More generally, bounds on the SOP with respect to all possible joint distributions are derived in [17].

One particularly interesting result in [17] is that the SOP in the best case can be equal to the outage probability due to the fading of the channel to the legitimate receiver, i.e., there exists a joint fading distribution that effectively hides the eavesdropper. The analysis in [17] is for a single-input single-output (SISO) scenario where all communication parties are equipped with only a single antenna. For a point-to-point transmission without eavesdropper, it was shown in [18,19] that it is possible to have a positive *zero*-outage capacity with multiple dependent fading channels. A natural question will be: can we extend the concept of *zero*-outage capacity, to the wiretap channel, namely, the zero-outage secrecy capacity (ZOSC)? By definition, zero-outage secrecy rate (ZOSR) is the secrecy rate of any achievable scheme which guarantees zero outage probability and ZOSC is the largest ZOSR. Please note that the ZOSC is an important performance metric because it describes the transmission rate of data which is guaranteed to be delivered reliably and confidentially for every channel use, i.e., for every set of channels to the legitimate receiver and to the eavesdropper. This implies that the eavesdropper does not obtain any information about the message and it also implies that the legitimate receiver is always able to correctly decode. Physical layer security mechanisms are the first line of defense [20] for future wireless communications systems. The ZOSC provides a very strong first line of defense. Therefore, the question remains, how much lower the SOP can be when the receiver and the eavesdropper have multiple observations and, in particular, if it is possible to obtain a positive ZOSC.

In this work, we answer the aforementioned question positively and show that there exist joint distributions which support a positive ZOSC. Note that the primary goal of the work is to show the existence of dependency structures which support positive ZOSC. We will not answer the question how to realize these dependency structures in practice. However, we point out that recent developments and progress in reconfigurable intelligent surfaces (RISs) [21] could enable to transform the propagation environment to the corresponding dependency structure.

Please also note that compared to our previous works [17,18], there are major differences in this work. In [17], we only consider the SISOSE model, i.e., every user only has a single antenna. Most importantly, with this model, it is not possible to achieve a positive ZOSR, which is the topic of the presented paper. Furthermore, it is not possible to simply apply the results from [17], since they only hold for two random variables. Since we now consider the multi-antenna case, we need to deal with more than two random variables. For a peaceful point-to-point system, we investigated the zero-outage capacity (ZOC) for systems with multiple receive antennas in [18]. However, in the previous paper, we only consider systems without the secrecy constraint and here, the secrecy constraint is considered. With this additional constraint, the rate/capacity expression is completely different. More specifically, to fulfill the secrecy constraint, wiretap coding must be used and then the secrecy rate expression in the additive white Gaussian noise (AWGN) channel has an additional negative term as the cost to fulfill the secrecy constraint. This negative term transforms the rate expression from symmetric into asymmetric in the channel gains. Due to this asymmetry, it is not possible to simply apply the results from [18] and it is therefore unclear, if the counter- and co-monotonicity of the dependence structure among the channel gains is optimal. These huge discrepancies are our main motivation to investigate the model considered in this paper and also the unique value of this work distinguished from the previous ones.

In particular, our contributions are summarized in the following.

- First, we consider a basic wiretap channel where there are two paths to a single legitimate receiver and one single path to a single eavesdropper. The channel gains are correlated slow fading and perfectly known at the receiver and eavesdropper, but unknown to the transmitter.
- Based on copulas, we derive an analytical solution for the ZOSR when Rayleigh fading is considered. In particular, the positive ZOSR is achieved by counter-monotonically distributed channel gains between the transmitter and the legitimate receiver. In contrast, the sum of the above two channel gains is co-monotonically distributed with respect to Eve’s channel gain.
- To gain a better understanding of the optimality of the dependency structure, we further transform the original ZOSC maximization problem into an equivalent form. Using the equivalent form, we propose an algorithm which efficiently solves the case where the channel gains are from finite alphabets. Interestingly, numerical results show that the optimal joint distribution of channel gains does not follow the aforementioned counter- and co-monotonicity relation.
- Then, we consider the generalization of the wiretap setup to multiple observations at Bob and Eve. We provide an algorithm to compute an achievable ZOSR and apply the rearrangement algorithm (RA) to solve the ZOSR problem for fading gains with continuous alphabets for a general number of observations.

Notation: Throughout this work, we use the following notation. Random variables are denoted in capital letters, e.g., *X*, and their realizations in small letters, e.g., *x*. Vectors are written in boldface letters, e.g., X$=(X_{1},\ldots ,X_{n})$. We use *F* and *f* for a probability distribution and its density, respectively. The expectation is denoted by E and the probability of an event by P. The uniform distribution on the interval [a,b] is denoted as U[a,b]. The normal distribution with mean μ and variance $\sigma^{2}$ is denoted as $N(\mu ,\sigma^{2})$. As a shorthand, we use ${\left[x\right]}^{+}=\max\left[x,0\right]$. The real numbers, non-negative real numbers, and extended real numbers are denoted by R, $R_{+}$, and $\bar{R}$, respectively. Logarithms, if not stated otherwise, are assumed to be with respect to the natural base.

## 2. System Model and Preliminaries

### 2.1. System Model

**Figure 1:**
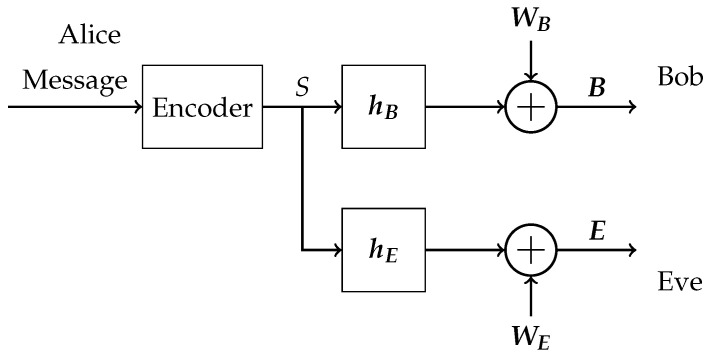
System model of the wiretap channel.

Throughout this work, we consider a slow-fading Gaussian wiretap channel [5], which is depicted in Figure 1.

The communication system consists of a single-antenna transmitter (Alice), a legitimate receiver (Bob), and a passive eavesdropper (Eve). Bob and Eve obtain $n_{B}$ and $n_{E}$ observations, respectively. These could stem from multiple antennas or from colluding eavesdroppers. Note that multiple colluding eavesdroppers and a single one with multiple antennas are equivalent. Alice encodes her messages, which she wants to transmit securely, and transmits symbols S∈C. Bob’s and Eve’s received signals are then, respectively, given as

(1) $$\begin{array}{cc} B & =h_{B}S+W_{B}\;, \end{array}$$

(2) $$\begin{array}{cc} E & =h_{E}S+W_{E}\;, \end{array}$$

where $h_{i}\in C^{n_{i}}$ and $W_{i}\in C^{n_{i}}$, with i∈{B,E}, denote the fading coefficients and the independent awgn with noise power $\sigma_{i}^{2}$, respectively. The transmission at Alice is subject to an average power constraint *P*, i.e.,

$$E_{}\left[{\left|S\right|}^{2}\right]\leq P\;.$$

The receiver signal-to-noise ratios (SNRs) are then given as $\rho_{B}=P/\sigma_{B}^{2}$ and $\rho_{E}=P/\sigma_{E}^{2}$ for Bob and Eve, respectively.

For one realization of the slow fading coefficients $h_{B}$ and $h_{E}$, the instantaneous secrecy capacity $C_{S}$ is given by ([22] Theorem 1 specialized to SIMOME wiretap channels)

(3) $$C_{S}={\left[\log_{2}\left(1+\sum_{i=1}^{n_{B}}X_{i}\right)-\log_{2}\left(1+\sum_{i=1}^{n_{E}}Y_{i}\right)\right]}^{+}$$

with the shorthands $X_{i}=\rho_{B}{\left|h_{B,i}\right|}^{2}$ and $Y_{i}=\rho_{E}{\left|h_{E,i}\right|}^{2}$.

In the following, we assume perfect channel-state information (CSI) at the receivers and perfect CSI about the main channel to Bob at the transmitter. However, only statistical CSI about the channel to Eve at Alice is assumed. For transmission, we consider a constant secrecy rate $R_{S}$. With this, it follows that a secrecy outage event happens, if the secrecy rate is greater than the instantaneous secrecy capacity, i.e., $C_{S}<R_{S}$. This leads to the definition of the SOP ε as ([2] Definition 5.1)

(4) $$\varepsilon =P\left(C_{S}<R_{S}\right)\;.$$

Based on the SOP, we can define the ε-outage secrecy capacity $R_{S}^{\varepsilon }$ as the highest rate, such that the SOP is at most ε, i.e.,

(5) $$R_{S}^{\varepsilon }=\underset{R\geq 0}{\sup}\left\{R\;|\;P\left(C_{S}<R\right)\leq \varepsilon \right\}\;.$$

A special case of the ε-outage secrecy capacity is the *zero*-outage secrecy capacity $R_{S}^{0}$, which denotes the highest rate, such there do not occur *any* secrecy outages.

### 2.2. Problem Formulation

We consider dependent fading channels, i.e., we have that the joint distribution of the fading channels does not factor into the product of marginal distributions, $F_{h_{B,1},\ldots ,h_{B,n_{B}},h_{E,1},\ldots ,h_{E,n_{E}}}\neq F_{h_{B,1}}\cdots F_{h_{B,n_{B}}}\cdot F_{h_{E,1}}\cdots F_{h_{E,n_{E}}}$. The joint distribution

$$F_{h_{B,1},\cdots ,h_{B,n_{B}},h_{E,1},\cdots ,h_{E,n_{E}}}$$

is unknown, however the marginals $F_{h_{i}}$ are assumed to be known. The marginals could be estimated from channel measurements of the corresponding point-to-point links.

With the above considerations, we can formulate the exact problem statement for the rest of this work. Our focus is on the ZOSC and we are interested in the question: *is it possible to obtain a positive ZOSC for certain dependent fading channels?* If yes, under what conditions and what are the values for common fading models?

For simplicity, we denote the configuration of the wiretap channel setup, which consists of $n_{B}$ observations at Bob, $n_{E}$ observations at Eve, by the notation $\left[n_{B},n_{E}\right]$-wiretap channel. After introducing the mathematical background, we consider the simple setup of the [2,1]-wiretap channel first ($n_{E}>1$ could correspond to the case of $n_{E}$ colluding eavesdroppers).

### 2.3. Mathematical Background

In order to answer the above questions, we need some mathematical background from copula theory [23], which we will introduce in the following.

The central elements of copula theory are *copulas*, which are defined as follows.

**Definition** **1** (Copula)**.** *A copula is an n-dimensional distribution function with standard uniform marginals.*

The practical relevance of copulas stems from Sklar’s theorem, which we restate in the following Theorem 1.

**Theorem** **1** (Sklar’s Theorem ([23] Theorem 2.10.9))**.** *Let $F_{X}$ be an n-dimensional distribution function with margins $F_{1},\ldots ,F_{n}$. Then there exists a copula C such that for all $x\in {\bar{R}}^{n}$,*

(6) $$F_{X}\left(x_{1},\ldots ,x_{n}\right)=C\left(F_{1}\left(x_{1}\right),\ldots ,F_{n}\left(x_{n}\right)\right)\;.$$

*If $F_{1},\ldots ,F_{n}$ are all continuous, then C is unique. Conversely, if C is a copula and $F_{1},\ldots ,F_{n}$ are distribution functions, then $F_{X}$ defined by (6) is an n-dimensional distribution function with margins $F_{1},\ldots ,F_{n}$.*

This theorem implies that copulas can be used to describe dependency structures among random variables, regardless of their marginal distributions. We can therefore separate the joint distribution into the dependency structure (described by the copula *C*) and the marginal distributions $F_{1},\ldots ,F_{n}$.

One of the best-known copulas is the independence copula $\prod \left(u_{1},\ldots ,u_{n}\right)=\prod_{i=1}^{n}u_{i}$, which describes independent random variables. Other relevant copulas are the Fréchet-Hoeffding bounds which we state in the following.

**Theorem** **2** (Fréchet-Hoeffding Bounds ([23] Theorem 2.10.12))**.** *Let C be a copula. Then for every $u\in {\left[0,1\right]}^{n}$*

(7) W(u)≤C(u)≤M(u)

*with*

(8) $$\begin{array}{cc} W(u) & =\max\left\{u_{1}+\cdots +u_{n}-n+1,0\right\}\;, \end{array}$$

(9) $$\begin{array}{cc} M(u) & =\min\left\{u_{1},\ldots ,u_{n}\right\}\;. \end{array}$$

In the case that n=2, *W* is a copula and two random variables whose joint distribution follows the copula *W* are called *countermonotonic*. The upper bound *M* is a copula for all *n* and random variables that follow *M* are called *comonotonic* [23]. The Fréchet-Hoeffding bounds are therefore a way to describe extreme positive and negative dependencies.

With these preliminary results, we are prepared to proceed and consider a simple scenario for the achievable ZOSC for the wiretap channel with two and one observation at the legitimate receiver and the eavesdropper, respectively.

## 3. Achievable ZOSC for the [2,1]-Wiretap Channel

First, we start with a simple scenario in which we consider two channels to Bob, modeled by the channel gains $X_{1}$ and $X_{2}$, and one channel to Eve, modeled by the channel gain *Y*. We consider the case with coherent combining at Bob with secrecy rate $R_{S}$. As a motivating example, let us consider Rayleigh fading on all channels, such that the channel gains to Bob are both standard exponential, i.e., $X_{1},X_{2}∼\mathrm{Exp}\left(1\right)$. The channel gain to Eve is also exponentially distributed but with mean μ, i.e., Y∼Exp(1/μ).

We will now show the surprising result that it is possible to achieve a positive ZOSR for dependent channels even without perfect channel-state information at the transmitter (CSI-T).

**Theorem** **3.** *The wiretap channel with two channels to Bob, both with marginal standard exponential distribution, and one channel to Eve exponentially distributed with mean μ≥0 can support a zero-outage secrecy rate*

(10) $$\mathrm{ZOSR}=\left\{\begin{array}{cc} 0 & \mu \geq 1\\ \log_{2}\left(1/\mu \right) & 1\geq \mu \geq \frac{1}{1+\ln(2)}\\ \min_{0\leq U\leq 1}\log_{2}\left(\frac{\ln(1/4-1/U^{2})-1}{\ln(1-U)\mu -1}\right) & \frac{1}{1+\ln(2)}>\mu \;. \end{array}\right.$$

**Proof.**  We start with the following first choice: $X_{1}$ and $X_{2}$ are counter-monotonic. This means that they are generated by one common uniform random variable U∼U[0,1] by

(11) $$X_{1}=F^{-1}\left(U\right),\;X_{2}=F^{-1}\left(1-U\right),$$

with inverse cumulative distribution function (CDF) $F^{-1}\left(z\right)=-\ln\left(1-z\right)$. For the sum of $X_{1}$ and $X_{2}$ we obtain

(12) $$S=X_{1}+X_{2}=-\ln\left(1-U\right)-\ln\left(U\right)=-\ln\left(U\left(1-U\right)\right).$$

The CDF of *S* is computed as

(13) $$F_{S}\left(s\right)=P\left(S\leq s\right)=P\left(U\left(1-U\right)\geq \exp\left(-s\right)\right).$$

Solving the inequality in (13) for *U* gives two roots $U_{1/2}=\frac{1}{2}\pm \sqrt{\frac{1}{4}-\exp\left(-s\right)}$. This allows to compute the CDF of *S* by

(14) $$F_{S}\left(s\right)=\int_{U_{1}}^{U_{2}}1\cdot ds=2\sqrt{\frac{1}{4}-\exp\left(-s\right)}=\sqrt{1-4\exp(-s)},\;\mathrm{for}\;s\geq 2\ln\left(2\right).$$

Next, we proceed with the second choice: *S* and *Y* are co-monotonic. In this case, the two random variables are generated by a common uniform random variable U∼U[0,1] as

(15) $$S=F_{S}^{-1}\left(U\right),\;Y=F_{Y}^{-1}\left(U\right)\;.$$

Therefore, the inverse CDF of *S* from (13) is given by

(16) $$F_{S}^{-1}\left(z\right)=\ln\left(\frac{4}{1-z^{2}}\right).$$

The SOP using the inverse CDF of *Y* with $F_{Y}^{-1}\left(z\right)=-\mu \ln\left(1-z\right)$ is computed as

(17) $$\begin{array}{cc} \varepsilon & =P(\log_{2}\left(1+S\right)-\log_{2}\left(1+Y\right)\leq R)\\ \; & =P\left(\log_{2}\left(\frac{1+F_{S}^{-1}\left(U\right)}{1+F_{Y}^{-1}\left(U\right)}\right)\leq R\right)\\ \; & =P\left(F_{S}^{-1}\left(U\right)-2^{R}F_{Y}^{-1}\left(U\right)\leq 2^{R}-1\right)\\ \; & =P\left(\ln\left(4\right)-\ln\left(1-U^{2}\right)+2^{R}\mu \ln\left(1-U\right)\leq 2^{R}-1\right)\\ \; & =P\left(\frac{{\left(1-U\right)}^{\mu 2^{R}}}{1-U^{2}}\leq \frac{1}{4}\exp\left(2^{R}-1\right)\right). \end{array}$$

Since we are interested in the ZOSR, the inequality inside (17) should not hold true for all 0≤U≤1. Therefore, the maximum ZOSR *R* has to fulfill

(18) $$\min_{0\leq U\leq 1}\frac{{\left(1-U\right)}^{\mu 2^{R}}}{1-U^{2}}\geq \frac{1}{4}\exp\left(2^{R}-1\right).$$

Solving (18) with respect to *R* gives the following characterization of the ZOSR

(19) $$\mathrm{ZOSR}=\min_{0\leq U\leq 1}\log_{2}\left(\frac{\ln(1-U^{2})-1-\ln4}{\mu \ln(1-U)-1}\right).$$

If μ≥1 the RHS in (19) is zero or negative. This indicates that the achievable ZOSR is zero if μ≥1. For $\mu \geq \frac{1}{1+\ln(2)}$ the RHS in (19) is monotonically decreasing with *U* and the minimum value is obtained for U→1. In this case, we have

(20) $$\mathrm{ZOSR}=\lim_{U\to 1}\log_{2}\left(\frac{\ln(1-U^{2})-1-\ln4}{\mu \ln(1-U)-1}\right)=\log_{2}\left(1/\mu \right)\;\mathrm{for}\;\;\mu \geq \frac{1}{1+\ln(2)}.$$

For $0\leq \mu <\frac{1}{1+\ln(2)}$ the RHS in (19) has a minimum within 0≤U≤1 and the ZOSR has to be evaluated via (19).    □

**Remark** **1.** *It can be observed that the channel to Eve should be worse on average compared to the channels to Bob, i.e., μ<1, in order to get a positive ZOSR. This can be interpreted as the advantage of the legitimate link over the eavesdropper’s link.*

We can easily extend the above scenario to the case where the eavesdropper has two observations, which both have an exponential marginal distribution with mean μ. For this extension, we have the following result on the achievable ZOSR.

**Corollary** **1.** *The wiretap channel with two channels to Bob, both with marginal standard exponential distribution and two channels to Eve, both with marginal exponential distribution with mean μ≥0 can support a zero-outage secrecy rate*

(21) $$\mathrm{ZOSR}={\left[\log_{2}\left(1+2\ln\left(2\right)\right)-\log_{2}\left(1+2\ln\left(2\right)\mu \right)\right]}^{+}\;.$$

**Proof.**  The proof follows similar lines as the proof of Theorem 3.    □

We conjecture that the dependency structure which leads to the achievable ZOSR in Theorem 3 is optimal. The intuition behind the result in Theorem 3 is to separate the two dependencies, the one between the legitimate channel gains $X_{1}$, $X_{2}$, and the other between the resulting legitimate joint channel gains $S_{X}=X_{1}+X_{2}$ and the eavesdropper channel gain *Y*. We know from [24] that counter-monotonicity of $X_{1}$ and $X_{2}$ is the best case for the ZOC. The resulting sum $S_{X}$ and the observation at Eve *Y* should be co-monotonic as will be shown in Lemma 1. Since a converse is not available yet, we proceed with the analysis of the ZOSR in the case of discrete alphabets.

## 4. An Equivalent Outage Problem Formulation for the [2,1]-Wiretap Channel

In this section, we first formulate equivalent expressions of the ZOSC for both cases where the channel gain is either continuous- or finite- alphabet. Then we solve the case in which the channel gain has finite alphabets from this equivalent expression.

An optimization problem to solve the ZOSC with continuous alphabet can be equivalently formulated as follows:

(22) $$\begin{array}{cc} P0:\;\underset{f_{X_{1}X_{2}Y}\in F}{\sup}\;\underset{x_{1},x_{2},y\in \mathrm{supp}\left(f_{X_{1}X_{2}Y}\right)}{\inf} & \;\log_{2}\left(\frac{1+x_{1}+x_{2}}{1+y}\right) \end{array}$$

(23) $$\begin{array}{cc} \;s.t.\; & \;f_{X_{1}X_{2}Y}\left(x_{1}^{*},x_{2}^{*},y^{*}\right)>0\\ \; & \;f_{X_{1}X_{2}Y}\left(x_{1},x_{2},y\right)\;\mathrm{is}\;\mathrm{cont}.\;\mathrm{in}\;\mathrm{the}\;r-\mathrm{neighborhood}\;\mathrm{of} \end{array}$$

(24) $$\begin{array}{cc} \; & \;(x_{1}^{*},x_{2}^{*},y^{*})\;\mathrm{for}\;\mathrm{some}r>0, \end{array}$$

where a feasible set of joint probability density functions F is defined as follows

(25) $$\begin{array}{cc} F:=\{f_{X_{1}X_{2}Y}\left(x_{1},x_{2},y\right)|\; & f_{X_{1}X_{2}Y}\left(x_{1},x_{2},y\right)=0,\mathrm{if}\;x_{1}+x_{2}<y, \end{array}$$

(26) $$\begin{array}{cc} \; & \int_{X_{2}\times Y}f_{X_{1}X_{2}Y}\left(x_{1},x_{2},y\right)dx_{2}dy={\tilde{f}}_{X_{1}}\left(x_{1}\right), \end{array}$$

(27) $$\begin{array}{cc} \; & \int_{X_{1}\times Y}f_{X_{1}X_{2}Y}\left(x_{1},x_{2},y\right)dx_{1}dy={\tilde{f}}_{X_{2}}\left(x_{2}\right), \end{array}$$

(28) $$\begin{array}{cc} \; & \int_{X_{1}\times X_{2}}f_{X_{1}X_{2}Y}\left(x_{1},x_{2},y\right)dx_{1}dx_{2}={\tilde{f}}_{Y}\left(y\right)\}, \end{array}$$

where (25) is to avoid non-positive secrecy rate, (26)–(28) are used to guarantee that the marginals are fixed as the given ones ${\tilde{f}}_{X_{1}}\left(x_{1}\right)$, ${\tilde{f}}_{X_{2}}\left(x_{2}\right)$, and ${\tilde{f}}_{Y}\left(y\right)$; $\left(x_{1}^{*},\;x_{2}^{*},\;y^{*}\right)$ is the tuple optimizing (22) while fulfilling (23); (23) and (24) together are to guarantee that $\left(x_{1}^{*},\;x_{2}^{*},\;y^{*}\right)$ occurs with a non-zero probability. In particular, a singular point in the probability density function (PDF) has a zero probability.

However, solving the functional optimization problem **P0** is involved. Therefore, instead of directly solving **P0**, in the following, we first consider the case with discrete channel gains, which can be obtained from the continuous one by quantization. Afterward, we propose to use the rearrangement algorithm to solve the ZOSR.

### Discrete Alphabets

We will now take a closer look at a simplified example where $X_{1}$, $X_{2}$, and *Y* are discrete random variables. In the simplest case, all of them are binary and either 0 or 1. The marginal distributions are again fixed and known. This yields the combinations and resulting secrecy capacities that are listed in Table 1. The joint probabilities of $\left(X_{1},X_{2},Y\right)$ for each combination are given in the last column as $F_{X_{1},X_{2},Y}$. First, since we want to achieve a positive ZOSC, we need to have zero probability for all combinations at which the secrecy capacity is zero. This leaves us with three degrees of freedom *a*, *b*, and *c*, as listed in Table 1.

From the fixed marginal distributions, we get the following conditions

(29) $$\begin{array}{cc} F_{X_{1}}\left(0\right) & =a\;, \end{array}$$

(30) $$\begin{array}{cc} F_{X_{2}}\left(0\right) & =b\;, \end{array}$$

(31) $$\begin{array}{cc} \;\;\;\;F_{Y}\left(0\right) & =a+b+c\;, \end{array}$$

which in turn yields

$$c=F_{Y}\left(0\right)-F_{X_{1}}\left(0\right)-F_{X_{2}}\left(0\right)\;.$$

Since *c* describes a joint probability, it needs to be non-negative and, therefore, the joint distribution in Table 1 is only valid for

(32) $$F_{Y}\left(0\right)\geq F_{X_{1}}\left(0\right)+F_{X_{2}}\left(0\right)\;,$$

which is equivalent to

(33) $$E_{}\left[X_{1}\right]\;+\;E_{}\left[X_{2}\right]\geq 1+E_{}\left[Y\right]$$

in terms of the expected values. The ZOSC is then equal to $\left(\log_{2}\left(3\right)-\log_{2}\left(2\right)\right)\;\approx 0.585$.

In order to extend the above discussion to cases with general finite alphabet sizes, we first define two sets as follows:

(34) $$\begin{array}{cc} J:= & \left\{j:\;\;\log_{2}\left(g\left(E_{j}\right)\right)\leq 0,\;whereg\left(E_{j}\right):=\frac{1+E_{j,1}+E_{j,2}}{1+E_{j,3}}\right\}, \end{array}$$

(35) $$\begin{array}{cc} \;L:= & [1,|M{|}^{3}]\backslash J, \end{array}$$

where E is the |M|-ary expansion matrix, e.g., the rows of E in order are as $\left(x_{1},x_{2},y\right)=\left(0,0,0\right),\;\left(0,0,1\right)$,⋯,(0,0,|M|−1),(0,1,0),⋯,(|M|−1,|M|−1,|M|−1), if M={0,1,2,⋯,|M|−1}. We then define a constant binary *marginalization matrix* $A\in {\left[0,1\right]}^{3|M|\times {\left|M\right|}^{3}}$, while the terminology comes from the fact that each row on the left hand side in the following expression

(36) $$\begin{array}{c} Ap=P \end{array}$$

is a marginalization to derive the marginal probability from the joint probability mass function (PMF), where the *j*-th entry of p is defined as follows

(37) $$\begin{array}{c} p_{j}:=E\left[{𝟙}_{E_{j}}\right], \end{array}$$

which is the probability that the *j*-th row of E happens, $j={1,\cdots ,|M|}^{3}$, P is a predefined marginal probability vector, which can be explicitly shown as follows:

(38) $$\begin{array}{c} P:={\left[P_{1}^{T},\;P_{2}^{T},\;\ldots ,\;P_{\left|M\right|}^{T}\right]}^{T}, \end{array}$$

where $P_{k}^{T}:=\left[P_{X_{1}}\left(k-1\right),\;P_{X_{2}}\left(k-1\right),\;P_{y}\left(k-1\right)\right],\;k=1,\ldots ,\left|M\right|$. Note that A is known due to the defined structure of E. Note also that $A_{i,j}=0$, if j∈J and then $p_{j}$ will not be used in calculating the marginal probability $P_{i}$, where $P_{i}$ is the *i*-th row of P, i=1,⋯,3|M|. Based on the above definitions, we can formulate the following optimization problem to derive the ZOSC:

(39) $$\begin{array}{cc} P1:\;\max_{\left\{p_{\ell }\right\}}\min\; & \;\log_{2}g\left(E_{\ell }\right) \end{array}$$

(40) $$\begin{array}{cc} \;s.t. & \;Ap=P \end{array}$$

(41) $$\begin{array}{cc} \; & \;\sum_{\ell \in L}p_{\ell }=1 \end{array}$$

(42) $$\begin{array}{cc} \; & \;0\leq p_{\ell }\leq 1,\;\ell \in L, \end{array}$$

where the objective function is to maximize the minimum instantaneous secrecy rate derived from all combinations of channel gains that have non-zero probabilities, by finding the PMF which fulfills the marginal probability constraint (40) and the feasibility constraints of the PMF (41) and (42). Note that $p_{l}=0,$ if $l\in L^{c}$. Note also that we do not need to take $l\in L^{c}$ into account in the objective function, since those $E_{l}$ with $l\in L^{c}$ will happen with probability zero, and do not affect the ZOSC.

Because $E_{l}$ is an implicit function of ${\left\{p_{l}\right\}}_{l\in L}$, we transform **P1** in the following equivalent form. We first re-order the rows of E such that values of the entries in ${\left\{\log_{2}g\left(E_{\pi_{i}}\right)\right\}}_{i\in L}$ are in an increasing manner, where the PMF defined in (37) becomes $\tilde{p}:=\left[p_{\pi_{1}},\;p_{\pi_{2}},\;\cdots ,p_{\pi_{\left|L\right|}}\right]$. The re-ordered E is defined as $\tilde{E}$. Note that this re-ordering does not change the ZOSC since the joint distribution of the channel gains remains unchanged but just the indexing is changed. Then the equivalent problem when the ZOSC is non-zero, is described as follows:

(43) $$\begin{array}{cc} \;P1{}^{\prime }:\;\min & \;L \end{array}$$

(44) $$\begin{array}{cc} s.t. & \;\tilde{A}\tilde{\tilde{p}}=\tilde{P} \end{array}$$

(45) $$\begin{array}{cc} \; & \;\sum_{i\in \tilde{L}}p_{\pi_{i}}=1 \end{array}$$

(46) $$\begin{array}{cc} \; & \;0<p_{\pi_{i}}<1,\;i\in \tilde{L} \end{array}$$

(47) $$\begin{array}{cc} \; & \;3|M|\leq L\leq |L|, \end{array}$$

where $\tilde{A}$ and $\tilde{P}$ are column-wise and row-wise re-ordered from A and P, respectively, due to the re-ordering of the rows of E, $\tilde{\tilde{p}}:={\left\{p_{\pi_{i}}\right\}}_{i\in \tilde{L}}$, $\tilde{L}:=\left\{|L|-L+1,\cdots ,|L|\right\}$. Note that $|\tilde{L}|=L$, as the number of non-zero probabilities of the instantaneous secrecy rate, is lower bounded by the number of rows in $\tilde{A}$, which results in the first inequality in (47). Otherwise, (44) will be over-determined. Please note that it may be possible that **P1** gives us the ZOSC as 0 and then there is no feasible solution from **P1${}^{\prime }$**.

The formulation of **P1${}^{\prime }$** can be explained intuitively as follows: ZOSC increases with decreasing $|\tilde{L}|=L$ due to the fact that values in ${\left\{\log_{2}g\left(E_{\pi_{i}}\right)\right\}}_{i\in \tilde{L}}$ are ordered in an increasing manner and if we decrease *L*, the number of the smallest |L|−L instantaneous secrecy rates being removed increases, which increases the ZOSC, as shown in (39). Then the smallest *L*, namely $L^{*}$, gives us the ZOSC as follows:

(48) $$\begin{array}{c} \mathrm{ZOSC}=\log_{2}g\left({\tilde{E}}_{\left|L\right|-L^{*}+1}\right). \end{array}$$

We define the ZOSC based on ${\left\{p_{l}\right\}}_{l\in L}$ as follows.

**Definition** **2** (ZOSR and ZOSC)**.** *Any feasible ${\left\{p_{l}\right\}}_{l\in L}$ results in an achievable ZOSR. The largest achievable ZOSR is the ZOSC.*

Based on Definition 2 and given a PMF ${\left\{p_{l}\right\}}_{l\in L}$ which is feasible to (40)–(42), we can try to increase the achievable ZOSR as follows: We first define $\tilde{l}:=\arg\min_{l\in L}g\left(E_{l}\right)$. Then let the $\tilde{l}$-th column of A as a zero vector. After that, we solve the corresponding **P1** again. This means that we re-allocate the non-zero probabilities to the instantaneous secrecy rates except the smallest one. If there exists a feasible PMF $\left\{p_{l}^{\prime }\right\}$ such that the resulting achievable ZOSR is larger than $\ln g(E_{\tilde{l}})$, then $\frac{1}{2}\log_{2}g\left(E_{\tilde{l}}\right)$ can not be the ZOSC. In contrast, if there does not exist a feasible $\left\{p_{l}^{\prime }\right\}$, then $\frac{1}{2}\log_{2}g\left(E_{\tilde{l}}\right)$ is the ZOSC. Based on these steps, we solve **P1${}^{\prime }$** alternatively by using the following finite deterministic Algorithm 1. Note that even if Ap=P is under-determined, after considering the constraints $0\leq p_{j}\leq 1,\;j=1,\;\cdots ,\;{\left|M\right|}^{3}$ and $\sum_{j=1}^{{\left|M\right|}^{3}}p_{j}=1$, the overall problem can also have no solution, depending on the initial values of P.

When constructing an algorithm to solve **P1${}^{\prime }$**, we should preset those joint probabilities whose corresponding instantaneous secrecy rates are the smallest to zero, such that the length of $\tilde{\tilde{p}}$ is the same as the number of rows of A. Then we gradually increase the length of $\tilde{\tilde{p}}$, solve the constrained linear system, and see if we can have a feasible solution and then we stop.

**Figure 2:**
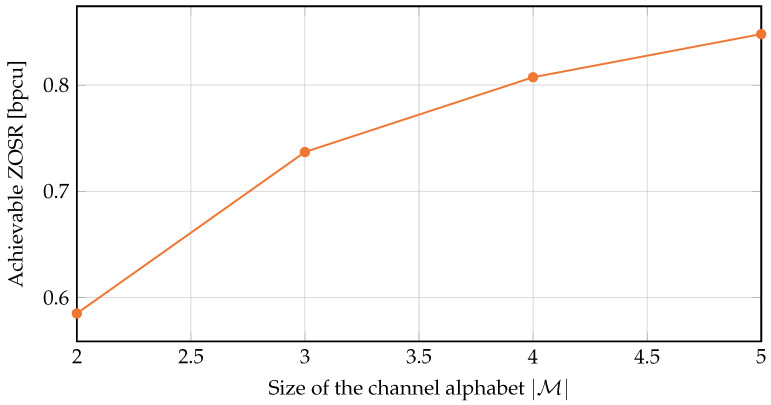
ZOSCs of the following alphabet sets M: {0,1}, {0,1,2}, {0,1,2,3}, and {0,1,2,3,4}.

Based on Algorithm 1, we can obtain the ZOSC as illustrated in Figure 2, where we consider the following marginal distributions:

- M={0,1}: $P_{X_{1}}\left(0\right)=0.2,\;P_{X_{2}}\left(0\right)=0.1,\;P_{Y}\left(0\right)=0.7$.
- M={0,1,2}: $P_{X_{1}}\left(0\right)=0.2,\;P_{X_{1}}\left(1\right)=0.3,P_{X_{2}}\left(0\right)=0.1,\;P_{X_{2}}\left(1\right)=0.2,\;P_{Y}\left(0\right)=0.7,\;P_{Y}\left(1\right)=0.1$.
- M={0,1,2,3}: $P_{X_{1}}\left(0\right)=0.2,\;P_{X_{1}}\left(1\right)=0.3,\;P_{X_{1}}\left(2\right)=0.1,P_{X_{2}}\left(0\right)=0.1,\;P_{X_{2}}\left(1\right)=0.2,\;P_{X_{2}}\left(2\right)=0.1,\;P_{Y}\left(0\right)=0.7,\;P_{Y}\left(1\right)=0.1,\;P_{Y}\left(2\right)=0.1$.
- M={0,1,2,3,4}: $P_{X_{1}}\left(0\right)=0.2,\;P_{X_{1}}\left(1\right)=0.3,\;P_{X_{1}}\left(2\right)=0.1,\;P_{X_{1}}\left(3\right)=0.1,\;P_{X_{2}}\left(0\right)=0.1,\;P_{X_{2}}\left(1\right)=0.2,\;P_{X_{2}}\left(2\right)=0.1,\;P_{X_{2}}\left(3\right)=0.05,\;P_{Y}\left(0\right)=0.7,\;P_{Y}\left(1\right)=0.1,\;P_{Y}\left(2\right)=0.1,\;P_{Y}\left(3\right)=0.04$.

In the following we prove the optimality of reaching ZOSC.
**Algorithm 1** Solve globally optimal ZOSC with channel gains from finite alphabet M0. Initialize the marginal distributions $P_{X_{1}}\in R_{+}^{|M|\times 1},\;P_{X_{2}}\in R_{+}^{|M|\times 1},\;P_{Y}\in R_{+}^{|M|\times 1}$ and define $P:={\left[P_{X_{1}}^{T},\;P_{X_{2}}^{T},\;P_{Y}^{T}\right]}^{T}$.1. Construct the |M|-ary expansion matrix E, where each row of E is a tuple $\left(x_{1},x_{2},y\right)\in M^{3}$ (the 1st row is (0,0,0) and the proceedings follow an increasing order with respect to the |M|-ary expansion).2. Construct $A\in {\left[0,1\right]}^{3|M|\times {\left|M\right|}^{3}}$, where $A_{i,j}=0$, if by the *j*-th row of E, $x_{1}+x_{2}\leq y$ and $p_{j}$ is not used in calculating the marginal probability $P_{i}$, where $P_{i}$ is the *i*-row of P, i=1,⋯,3|M|, $j={1,\cdots ,|M|}^{3}$. Define L=3|M|.3. Reorder A and E as $\tilde{A}$ and $\tilde{E}$, respectively, such that ${\left\{\frac{1}{2}\ln g\left(\tilde{E_{i}}\right)\right\}}_{i\in L}$ are in an increasing manner.**repeat**  4. Update $\tilde{A}$: set $\tilde{L}-L$ columns of $\tilde{A}$ as a zero vectors, where the indices of those columns correspond to the rows of $\tilde{E}$ having the smallest rates.  5. Solve $\tilde{A}\tilde{\tilde{\tilde{p}}}=\tilde{P}$, $0<{\tilde{\tilde{\tilde{p}}}}_{i}\leq 1$, $\sum p_{i}=1$, $i\in \tilde{L}$, by CVX.  6. Set L = L + 1.**until**$\tilde{A}\tilde{\tilde{\tilde{p}}}=\tilde{P}$ is feasible,7. ZOSC = $\min_{(x_{1},\;x_{2},\;y)\in F}\frac{1}{2}\left[\log_{2}\left(1+x_{1}+x_{2}\right)-\log_{2}\left(1+y\right)\right]$, where$F:=\{E_{j}|\;\;\mathrm{if}\;p_{j}>0,\;j=1\cdots ,|M{|}^{3}\}$.

**Theorem** **4.** *Algorithm 1 computes the ZOSC for discrete alphabets in a finite number of steps.*

**Proof.**  We use the following steps to prove. First, in order to obtain a positive ZOSC, a number of entries of the probabilities whose indices belonged to J have to be set to zero. Then we use the programming problem **P1** to determine the ZOSC. We further transform problem **P1** into the equivalent programming problem **P1${}^{\prime }$**. The implementation in Algorithm 1 efficiently solves **P1${}^{\prime }$**. Note that the greedy-like algorithm guarantees to find the ZOSC instead of only a ZOSR. This is because, in the equivalent problem **P1**, the secrecy rate is ordered and therefore, at each step the greedy algorithm in fact, accesses the global information to reach the global optimum. □

**Remark** **2.** *Due to the affine and linear properties in the set of constraints, we can easily extend the setting of* **P1${}^{\prime }$** *and Algorithm 1 to parallel channels.*

**Remark** **3.** When comparing secure systems to cases without secrecy constraint, we can observe an obvious difference in the optimal joint distribution. For the latter case, the ZOC happens when the channel gains are counter-monotonic. Intuitively, it avoids the cases in which the multiple channel gains are simultaneously small. However, for the ZOSC, we do not observe the counter- or co-monotonicity property on the channel gains from finite alphabets. In particular, by the numerical results, we can observe that we obtain the ZOSCs when $x_{1}=x_{2}=y$ for all |M|=2,3, and *4*, i.e., $\left(x_{1}^{*},x_{2}^{*},y^{*}\right)=\left(1,1,1\right),\;\left(2,2,2\right),\;$(3,3,3), and (4,4,4), respectively. These examples show that the relation between $X_{1},\;X_{2},$ and Y does not necessarily follow the rule in ZOC that $X_{1}$ and $X_{2}$ are counter-monotonic. The missing counter- or co-monotonicity property is mainly due to the missing symmetry of the objective function with respect to the random variables.

## 5. Positive ZOSR for [$n_{B}$,$n_{E}$]-Wiretap Channels

After exploring a particular dependency structure to achieve a positive ZOSR for $n_{B}=2$ and $n_{E}=1$ in Section 3, we will now generalize the approach to multiple observations at Bob and Eve.

First, recall that the secrecy capacity $C_{S}$ in this case is given by (3) as

(49) $$C_{S}=\log_{2}\left(\frac{1+S_{B}}{1+S_{E}}\right)\;,$$

where we introduce the shorthand notations $S_{B}=\sum_{i=1}^{n_{B}}X_{i}$ and $S_{E}=\sum_{i=1}^{n_{E}}Y_{i}$.

In order to specify an appropriate joint distribution for a positive ZOSR, we need the following observation.

**Lemma** **1.** *For fixed distributions of $S_{B}$ and $S_{E}$, the ZOSR in (49) is maximized for comonotonic $S_{B}$ and $S_{E}$, i.e., for*

(50) $$S_{B}=F_{S_{B}}^{-1}\left(U\right)\;\mathrm{and}\;S_{E}=F_{S_{E}}^{-1}\left(U\right),$$

*with*U∼U[0,1].

**Proof.**  Assuming fixed joint distributions of Bob’s and Eve’s channel gains, respectively, we obtain fixed distributions of $S_{B}$ and $S_{E}$. Based on (5), the ZOSR can then be formulated as

(51) $$\mathrm{ZOSR}=\underset{s}{\sup}\left\{s\;|\;P\left(\log_{2}\left(1+S_{B}\right)-\log_{2}\left(1+S_{E}\right)\leq s\right)=0\right\}\;.$$

We can rewrite this optimization problem as

(52) $$\mathrm{ZOSR}=\underset{s}{\sup}\left\{s\;|\;P\left({\tilde{S}}_{B}+{\tilde{S}}_{E}\leq s\right)=0\right\}\;,$$

where we use

$${\tilde{S}}_{B}=\log_{2}\left(1+S_{B}\right),\;{\tilde{S}}_{E}=-\log_{2}\left(1+S_{E}\right)\;.$$

Since the inner function is now a sum, we can apply ([25] Theorem 3.1) and derive that (52) is solved for counter-monotonic ${\tilde{S}}_{B}$ and ${\tilde{S}}_{E}$, i.e., for ${\tilde{S}}_{B}=F_{{\tilde{S}}_{B}}^{-1}\left(U\right)$ and ${\tilde{S}}_{E}=F_{{\tilde{S}}_{E}}^{-1}\left(1-U\right)$, with U∼U[0,1].

It is straightforward to see that $F_{{\tilde{S}}_{B}}^{-1}\left(U\right)=\log_{2}\left(1+F_{S_{B}}^{-1}\left(U\right)\right)$. For ${\tilde{S}}_{E}$, we get

(53) $$\begin{array}{cc} F_{{\tilde{S}}_{E}}\left(s\right)\; & =P\left(S_{E}\geq 2^{-s}-1\right) \end{array}$$

(54) $$\begin{array}{cc} \; & =1-F_{S_{E}}\left(2^{-1}-1\right) \end{array}$$

and thus

(55) $$F_{{\tilde{S}}_{E}}^{-1}\left(U\right)=-\log_{2}\left(1+F_{S_{E}}^{-1}\left(1-U\right)\right)\;.$$

Therefore, for counter-monotonic $\left({\tilde{S}}_{B},{\tilde{S}}_{E}\right)$, we get

$$\begin{array}{cc} F_{{\tilde{S}}_{B}}^{-1}\left(U\right) & =\log_{2}\left(1+F_{S_{B}}^{-1}\left(U\right)\right)\\ F_{{\tilde{S}}_{E}}^{-1}\left(1-U\right) & =-\log_{2}\left(1+F_{S_{E}}^{-1}\left(U\right)\right)\;, \end{array}$$

i.e., co-monotonic $\left(S_{B},S_{E}\right)$. □

Based on Lemma 1, we next fix the dependency structure in the following way, in order to achieve a positive ZOSR.

1. First, we find the joint distribution between Bob’s channels $X_{1},\ldots ,X_{n_{B}}$ that maximizes the ZOC. The simple reason behind this first step is that we cannot find a positive ZOSC, if we do not have a positive ZOC to the legitimate receiver. In fact, the ZOSC is upper bounded by the ZOC to Bob.
2. Next, we find the same dependency structure for Eve’s channels $Y_{1},\ldots ,Y_{n_{E}}$ that maximizes the ZOC. It may seem counter-intuitive to choose a joint distribution for which $S_{E}$ is always greater than a positive constant. However, the reasoning behind this particular choice is to balance the realizations of Eve’s channels such that only little probability mass is placed on high realizations of $S_{E}$. Otherwise, there could be a positive probability that $S_{E}>S_{B}$, which would result in a ZOSC of zero. It should be emphasized that this is a particular choice for this scheme and might not be the optimal dependency for the general case.
3. Finally, we set $S_{B}$ and $S_{E}$ as co-monotonic in order to maximize the ZOSR for fixed $F_{S_{B}}$ and $F_{S_{E}}$ as shown in Lemma 1.

It is apparent that the first two steps are difficult to solve in general [18]. However, there exists a RA [26] that can be used to approximate the ZOC along with the corresponding joint distribution. In the following, we apply the RA to solve the optimization problems

$$\min_{F_{X}:X_{i}∼F_{i}}F_{X_{1}+\ldots +X_{d}}^{-1}\left(\alpha \right)\;\mathrm{and}\;\max_{F_{X}:X_{i}∼F_{i}}F_{X_{1}+\ldots +X_{d}}^{-1}\left(\alpha \right)\;,$$

as shown in [26]. In particular, we apply the RA to solve Steps 1 and 2 from above.

The idea behind the RA is to find a multi-variate version of counter-monotonicity between multiple random variables [27]. Due to the numerical nature of the algorithm, we first need to introduce some required approximations and notations. Detailed explanations of the algorithm and individual steps, which are described in the following, can be found in [26].

Given a matrix $A\in R^{N\times n}$, we obtain the matrix $A_{\left(-j\right)}\in R^{N\times n-1}$ by deleting the *j*-th column $A_{\left(j\right)}$ from *A*. Each column of the matrix represents a random variable $X_{j}$ with given marginal distribution $F_{X_{j}}$. This is achieved by quantizing the CDF into *N* steps by the following two ways

$${\underline{a}}_{i,j}=F_{X_{j}}^{-1}\left(\frac{i-1}{N}\right)\;\mathrm{and}\;{\bar{a}}_{i,j}=F_{X_{j}}^{-1}\left(\frac{i}{N}\right),\;1\leq i\leq N\;.$$

This yields the matrices $\underline{A}$ and $\bar{A}$, respectively. The quantization ${\underline{a}}_{i,j}$ serves as a lower bound on $F_{X_{j}}$, while ${\bar{a}}_{i,j}$ is an upper bound. It can be seen that the quantization becomes closer to the original CDF with increasing *N*. Starting with the initial matrices $\underline{A}$ and $\bar{A}$, these matrices are rearranged iteratively such that in each step the *j*-th column $A_{\left(j\right)}$ is oppositely ordered to the row-wise sum of $A_{\left(-j\right)}$. This opposite rearrangement makes the random variables represented by $A_{\left(j\right)}$ and the sum of $A_{\left(-j\right)}$ countermonotonic. The rearrangement step is repeated for each column until convergence. The final output of the RA are the matrices ${\underline{A}}^{🟉}$ and ${\bar{A}}^{🟉}$ resulting from the rearrangement of $\underline{A}$ and $\bar{A}$, respectively.

Each row of ${\underline{A}}^{🟉}$ and ${\bar{A}}^{🟉}$ represents an *n*-dimensional point of the joint distribution function of $\left(X_{1},\ldots ,X_{n}\right)$. From this approximation of the optimal joint distribution, the final approximation of the ZOC is derived.

The algorithm was originally developed to calculate bounds on the distribution of functions of dependent risks [28] and it is applied in the area of actuarial science and quantitative risk management [26]. In communications, it has already been used to numerically approximate the best and worst case ergodic capacity for dependent fading channels [29].

The source code to reproduce all of the following numerical results can be found at [30]. The RA is implemented in Python [31] based on [32].

**Remark** **4.** *The idea of achieving a positive ZOC is closely connected to the concept of* joint mixability [27,33]. *If the fading gains would be jointly mixable, their sum would almost surely add up to a constant, i.e., the overall fading becomes deterministic. If this is possible for both Bob’s and Eve’s channels, the resulting secrecy capacity would be a constant, i.e., it corresponds to the ZOSC. Unfortunately, distributions with one-sided unbounded support can not be jointly mixable *([33] *Rem. 2.2*), *which includes most of the common fading gain distributions. It should be noted that the scheme described above only finds an achievable positive ZOSR, which might not be the maximum. When taking the joint distribution of all channel gains into account—without separately optimizing Bob’s and Eve’s joint distributions—there might arise a similar mixability structure where high realizations of $Y_{i}$ are compensated by high realizations of $X_{i}$, resulting in a constant secrecy rate.*

### Example: Rayleigh Fading

In order to demonstrate the effectiveness of the RA, we now consider the scenario of homogeneous Rayleigh fading, analogue to Section 3. In this case, all $n_{B}$ channel gains to Bob are distributed according to an exponential distribution with mean 1, i.e., $X_{i}∼\mathrm{Exp}\left(1\right)$ for all $i=1,\ldots ,n_{B}$. In order to reflect an SNR difference between Bob and Eve, we assume that the channel gains of Eve’s channels have mean μ, i.e., $Y_{i}∼\mathrm{Exp}\left(1/\mu \right)$ for all $i=1,\ldots ,n_{E}$.

**Figure 3:**
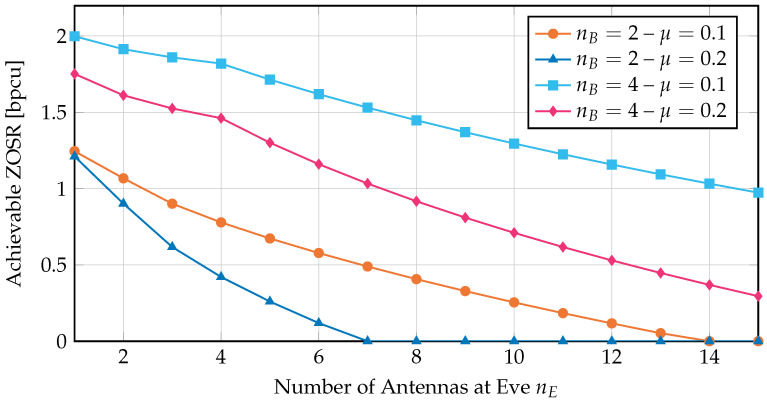
Achievable ZOSR for Rayleigh fading with $n_{B}$ antennas at Bob and $n_{E}$ antennas at Eve. The mean of Eve’s channel gains is μ. The results are calculated using the RA with $N=10^{4}$ quantization steps.

In Figure 3, we show the calculated ZOSRs over the number of Eve’s antennas $n_{E}$ using the RA with the scheme described above. The results are presented for multiple values of μ and $n_{B}$. The first expected observation is that the ZOSR decreases with an increasing number of antennas at Eve, while it increases when $n_{B}$ is increased. Similarly, if the channel quality of Eve’s channels μ improves, the achievable ZOSR decreases. Interestingly, the slope of decreasing ZOSR is steeper for $n_{E}>n_{B}$, which is particularly visible in Figure 3 for $n_{B}=4$. It can also be seen that the ZOSR is equal to zero above a certain number of antennas at Eve, depending on $n_{B}$ and μ. However, the results in Figure 3 demonstrate that it is generally possible to find joint distributions supporting a positive ZOSR by applying the RA. It should also be noted that this is not limited to the homogeneous case, but arbitrary marginal distributions are supported.

**Remark** **5.** *As described at the beginning of this section, the RA returns a lower and an upper bound on the exact value. The results in Figure 3 are obtained with $N=10^{4}$ quantization steps. With this number of quantization steps, the resulting bounds from the RA cannot be distinguished in Figure 3 and are therefore shown as one single curve.*

## 6. Conclusions and Future Work

In this paper, we consider the setting that there are two paths from the transmitter to the legitimate receiver and one path (or multiple co-monotonic paths) to the eavesdropper. We show that we can achieve a zero SOP for some positive secrecy rate by introducing a proper dependence structure among the fading gains of the three paths. Thereby, we can achieve a non-zero ZOSR. To better understand the underlying dependence structure, we further consider the case where the channel gains are from finite alphabets and systematically and globally solve the zero outage secrecy capacity. In order to obtain positive ZOSR, an advantage in terms of average channel gains must exist. In addition, the results are underlined with numerical constructions by the RA to solve the ZOSR for continuous channel gains. The generalized results to multiple observations at Bob and Eve indicate that positive ZOSR can be achieved if the legitimate link has an advantage in terms of the number of observations and the average channel gains.

For future work, we have the open problem to determine the global optimal dependency structure for the continuous distributions. We conjecture that the dependency structure that leads to Theorem 3 is the global optimal to maximize the ZOSR. Another open question is how to connect the model and results for the discrete alphabet case to the continuous distribution case. Furthermore, the construction of an improved algorithm for the multi-observation case based on joint mixability and tail distribution analyses is open for future work, too.

The last but not the least point for the future work is on how to practically implement the designed joint distribution. RIS [34], a recently hot research topic, shows the high possibility of manipulating the channels, artificially. However, the inter-relation between the fundamental properties of RIS and the designed joint distribution is still missing, which can be a critical issue to achieve our design goal discussed in this paper.

## Figures and Tables

**Table 1:** A binary example.

$X_{1}$	$X_{2}$	*Y*	Secrecy Capacity	$F_{X_{1},X_{2},Y}$
0	0	0	0	0
0	0	1	0	0
0	1	0	1	*a*
0	1	1	0	0
1	0	0	1	*b*
1	0	1	0	0
1	1	0	1.585	*c*
1	1	1	0.585	1−a−b−c

## Data Availability

The source code of all used algorithms and calculations is publicly available under the GPLv3 license at [30]. This allows reproducing all of the presented results.

## Abbreviations

The following abbreviations are used in this manuscript:

AWGN	additive white Gaussian noise
CDF	cumulative distribution function
CSI	channel-state information
CSI-T	channel-state information at the transmitter
PDF	probability density function
PMF	probability mass function
RA	rearrangement algorithm
RIS	reconfigurable intelligent surface
SISO	single-input single-output
SNR	signal-to-noise ratio
SOP	secrecy outage probability
ZOC	zero-outage capacity
ZOSC	zero-outage secrecy capacity
ZOSR	zero-outage secrecy rate
